# Stability under humidity, UV-light and bending of AZO films deposited by ALD on Kapton

**DOI:** 10.1038/s41598-019-54451-0

**Published:** 2019-11-29

**Authors:** A. C. Marques, J. Faria, P. Perdigão, B. M. M. Faustino, Riina Ritasalo, Katiuscia Costabello, R. C. da Silva, I. Ferreira

**Affiliations:** 1 CENIMAT/I3N, Departamento de Ciência dos Materiais, Faculdade de Ciências e Tecnologia, Universidade Nova de Lisboa, Caparica, 2829-516 Portugal; 2 Picosun Oy Masalantie 365, FI-02430 Masala, Finland; 3 GRINP Srl, via De Francisco 123, 10036 Settimo T.se, TO Italy; 4 IPFN-IST/UL, Instituto de Plasmas e Fusão Nuclear, Instituto Superior Técnico, Universidade de Lisboa, Estrada Nacional 10, 2695-066 Bobadela, Portugal

**Keywords:** Sensors and biosensors, Devices for energy harvesting

## Abstract

Aluminium doped zinc oxide (AZO) films were grown by Atomic Layer Deposition (ALD) on yellow Kapton and transparent Kapton (type CS) substrates for large area flexible transparent thermoelectric applications, which performance relies on the thermoelectric properties of the transparent AZO films. Therefore, their adhesion to Kapton, environmental and bending stability were accessed. Plasma treatment on Kapton substrates improved films adhesion, reduced cracks formation, and enhanced electrical resistance stability over time, of importance for long term thermoelectric applications in external environment. While exposure to UV light intensity caused the films electrical resistance to vary, and therefore their maximum power density outputs (0.3–0.4 mW/cm^3^) for a constant temperature difference (∼10 °C), humidity exposure and consecutive bending up to a curvature radius above the critical one (∼18 mm) not. Testing whether the films can benefit from encapsulation revealed that this can provide extra bending stability and prevent contacts deterioration in the long term.

## Introduction

This work demonstrates the stability of AZO films deposited by ALD on Kapton when exposed to external elements – humidity, UV light and bending, – in view of its potential use in large area flexible electronic devices over extended periods of time.

Atomic layer deposition (ALD) is a gas phase thin film deposition method, known to be good for surface conformity and precise atomic level control of film composition and thickness through surface reactions, providing uniform dopant distribution. The deposition can be done in large areas and at relatively low temperatures (200 °C or lower), making it compatible with polymeric substrates (e.g. as Kapton polyimide) commonly used for e.g. Organic Light-Emitting Diodes (OLEDs)^1^, energy harvesting devices in large smart windows^2^ and thermoelectric touch sensors^3^. Kapton is an adequate substrate since it is transparent and has softening temperatures (Tg > 360 °C) that allow ALD^4^. Fabrication of metal oxide films by ALD has emerged in the last decade as their properties are competitive with others made by a variety of processes^5–7^. Films made by stacking ZnO and Al_2_O_3_ layers grown by ALD revealed similar electro-optical properties to AZO grown by sputtering and do not require post-annealing^8,9^. Recently, H.-W. Park deposited conformal high quality ZnO/Al_2_O_3_ stacked multilayers on transparent nanostructures with improved optical and electrical properties^10^. Hence, and because of the similar properties ALD ZnO/Al_2_O_3_ multilayered films are often and hereon referred to as AZO films. Prior to the AZO stack deposition, a 10 nm thick Al_2_O_3_ layer is used as an interface to avoid diffusion of chemical species to and from the polymeric substrates, improve adhesion and reduce cracks formation. Alumina adhesion to polymer substrates is remarkable due to the covalent bonding^11^. Nonetheless, a plasma treatment of polymeric substrates is also frequently used to improve adhesion and performance of the grown films^12^. In this work, plasma treatment of some Kapton substrates was performed prior to the ALD deposition, aiming at further improving adhesion. As it will be shown in the following, the long term performance and stability of the films under UV-light was concurrently improved. Moreover, the ALD AZO films proved to have low sensitivity to humidity and bending. Encapsulation did not hinder the performance of the devices but improved stability while protecting the electrodes (usually very sensitive to oxidation and moisture).

## Experimental Details

AZO films were grown by ALD onto Al_2_O_3_ coated 25 μm thick Kapton substrates (CS and Yellow types), with and without previous plasma treatment. The Al_2_O_3_ coating was grown at 50 °C to a thickness of 10 nm. The functionalization of Kapton surfaces prior to ALD deposition aimed at improving adhesion of deposited thin layers through two type of plasma treatments: He/O_2_ (T1) and He/N_2_ (T2), both performed on a GRINP PLAFilm HT 600 machine that creates plasmas at atmospheric pressure by dielectric barrier discharge (DBD). Mixed gas-plasmas of He/O_2_ and He/N_2_ were produced at room temperature with a mixing ratio of 4:1 and of 4:3, respectively. The source-to-sample distance was 1 mm and the scan speed 33 mm/s, with the plasma power set to 2000 W.

The ALD was performed with PICOSUN P-300B ALD system under N_2_ atmosphere at a pressure inferior to 1 mbar and a substrate temperature of 200 °C. The multilayered films were grown on top of the 10 nm Al_2_O_3_ interfacial layer in the sequence: nominal 22 nm thick ZnO followed by 0.5 nm Al_2_O_3_ until the desired nominal thickness, of 100 nm or 460 nm was reached. Hence, the number of precursor cycles for the referred thicknesses and an Al content of 1.5–4%, totalized 4 and 20 master cycles, respectively. The precursors used for deposition of Al_2_O_3_ and ZnO layers were trimethyl-aluminum and diethyl-zinc with H_2_O. The composition and thicknesses of the AZO films deposited by ALD on Kapton substrates were measured by Rutherford Backscattering Spectroscopy (RBS) using a 2 MeV ^4^He^+^ beam. RBS spectra were collected at 140° and 165° scattering angles by means of Si-diode detectors of 16 keV energy resolution, and analysed with the help of the RUMP code^13^. Some key features of the RBS technique applied to thin films are the ability to determine elemental composition and thickness of the layers. Briefly, the position of the spectral features of a RBS spectrum correlate with the masses of the scattering centres (and so with the contributing elements), its height with the amount of the contributing elements present (*i*.*e*. the corresponding concentrations) and its width or energy extent with the physical thickness that those elements take.

Characterization of the surface morphology was performed with a Hitachi S2400 Scanning Electron Microscope (SEM) operated in Secondary Electron Imaging (SEI) mode and with the Atomic Force Microscope (AFM) of WITec Alpha 300 RAS confocal spectrometer. The cantilever was operated with an Al coated probe in AC mode at 75 kHz and constant load of 2.8 N/m. Lateral and depth resolutions were 1 nm and 0.3 nm, respectively.

AZO films were submitted to variable relative humidity (%) inside a glove box equipped with a cold steam sprayer and a RS Pro 408–6109 humidity electronic sensor, providing an uncertainty of ±5% in the range 25% to 80%, and ±10% out of that range. The relative humidity inside the glove box was first raised from 50% to 100% in steps of 5% (cycle 1) and next lowered by venting the box (cycle 2). Cycles 1 and 2 lasted for ∼104 min and ∼120 min, respectively. The influence of UV-light exposure was carried out in a UV-light box with a lamp emitting a dominant wavelength of 288 nm (4.31 eV), enabling photo excitation of AZO electrical carriers. For comparison, the AZO films were also submitted to sunlight UV radiation.

Electric and thermoelectric characterizations were performed with a home-made setup^14^, imposing a thermal gradient (ΔT) in the plane of film and substrate using two TEC1–12707 Peltier modules, connected to independent power sources. The Seebeck coefficient was determined by imposing a ∆T that was monitored by a FLIRA310 thermal camera, while the thermoelectric voltage (ΔV) was measured using an Agilent 34420 A nano-voltmeter, using in-plane Al electrodes deposited by thermal evaporation on top of the AZO film, with a size of 3 × 6 mm^2^ and 3 mm separation. Seebeck coefficient was obtained from the slope of the plot ΔV vs. ΔT shown in (Fig. S1), subsequently enabling the calculus of the power factor. A variable load resistance connected to the thermoelectric elements and ΔV measurements across terminals, from short-circuit to open-circuit conditions enable power output determination (P_out_ = I_out_ × V_out_). The AZO films resistance over time was continuously monitored by a Keysight 34972A LXI data acquisition unit connected to a computer with home-made software.

The transmittance measurements were performed in a JASCO V-770 spectrophotometer, in the wavelength range 190–2500 nm, in steps of 0.5 nm. The bending tests were performed with a home-made machine that measures the film electrical resistance as a function of the bending radius, calculated according to the International Electrotechnical Commission standard 47/2199/NP draft. Hence, a continuous resistance measurement profile is taken for each sample while bending.

## Results and Discussion

### Composition and morphology

**Figure 1:**
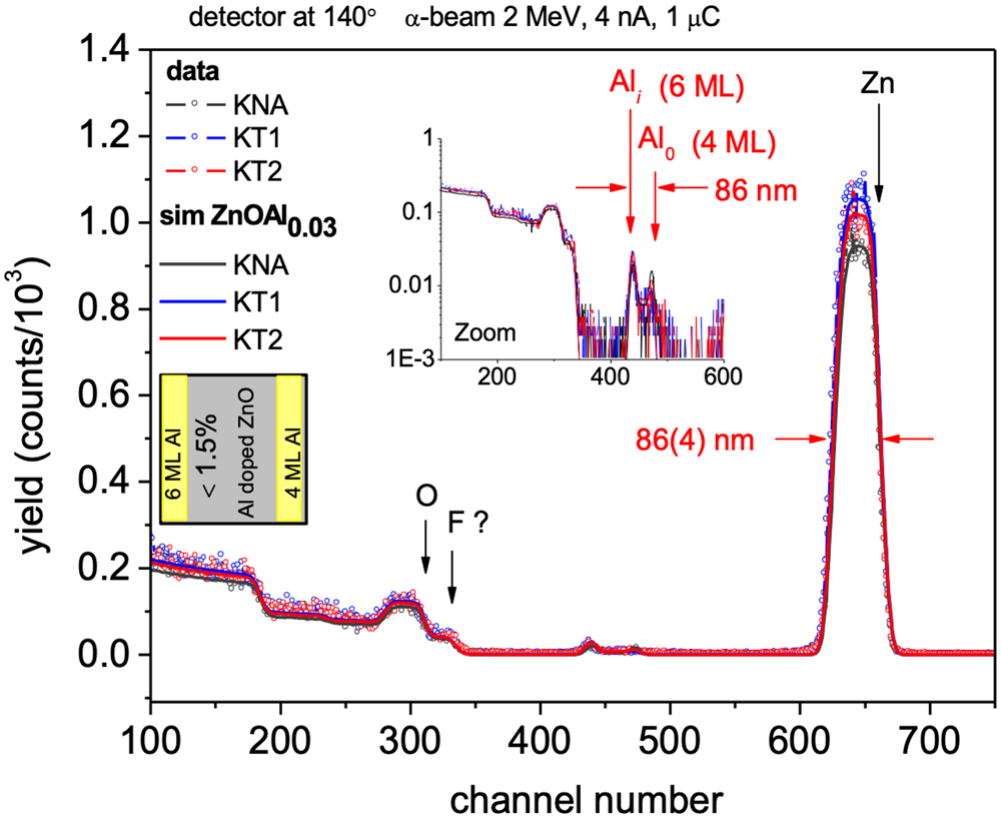
Experimental RBS spectra (open symbols) and simulations (full lines) from samples with nominal 90 nm AZO films + 10 nm Al_2_O_3_ buffer layer deposited by ALD onto Kapton CS substrates without and with plasma treatment of H_2_/O_2_ (T1) and He/N_2_ (T2).

Representative RBS spectra of AZO films deposited by ALD on untreated (KNA) and plasma treated Kapton CS substrates (with T1 and T2) are shown in Fig. 1. The full lines represent the simulations that best fit the experimental data, as calculated with the RUMP code^13^, allowing to extract the overall elemental compositions and thicknesses. These simulations were generated starting from a base model of a layered structure of equal Al_2_O_3_/ZnO double-layers with thicknesses and compositions equal to the nominal values of deposition. The substrates are composed of Carbon, Hydrogen and Nitrogen. Fluorine was also detected – its contribution to the RBS spectra is clearly pointed at, – a finding that is not totally unexpected, since DuPont Kapton is often coated with a fluoropolymer resin layer (e.g. FEP) for imparting heat sealability, moisture barrier protection, and chemical resistance enhancement^4^. The RBS spectra of the AZO samples show a structure consisting of Zn, Al and O profiles of similar energy widths, with two thin Al peaks separated in energy by a difference consistent with the energy widths of the Zn, Al and O profiles (*cf*. inset of Fig. 1). Accordingly, the full AZO films spread along (86 ± 4) nm. The two Al peaks limiting the Al profile in the RBS spectra correspond to the Al in the first Al_2_O_3_ layer of the stack (identified as Al_0_ in Fig. 1) and to the Al_2_O_3_ buffer layer between AZO and the substrate (A_*i*_ in Fig. 1). The higher intensity of the Al peak at lower energy (near channel 400) agrees with the significantly larger width of the buffer layer as compared with the Al_2_O_3_ layers in the stack. The overall amount of Al was found to be less than 1.5 at.%. This value is in agreement with EDS measurements. Notably the films thicknesses extracted from the simulations are remarkably the same for all analysed samples (*i*.*e*. KNA, T1 and T2) belonging to a common ALD run, indicating that the ALD process is reproducible and uniform in small areas without visible coating defects or folds. The AZO films thickness of (86 ± 4) nm measured through RBS is within the thickness range of 85–95 nm measured by optical ellipsometry.

The plasma treatment is known to change the surface morphology and create chemically active functional groups and radicals that are able to enhance the wettability and surface energy of Polymers^15,16^. For instance, hydroxyl and carboxyl groups (e.g. OH, −C=O, −COOH) for the He/O_2_ treatment. Such chemically active groups help forming affinity or chemical bonds with a coating material and thus adhesion is improved. Elemental composition of plasma-treated kapton film surface was previously addressed by N. Inagaki *et al*. and it was demonstrated that the plasma treatment led to incorporation of oxygen atoms rather than nitrogen atom^17^. The O/C atomic ratio of the plasma treated kapton film, determined by XPS in this work, revealed to be 1.9–2.3 times higher than that of the untreated Kapton.

**Figure 2:**
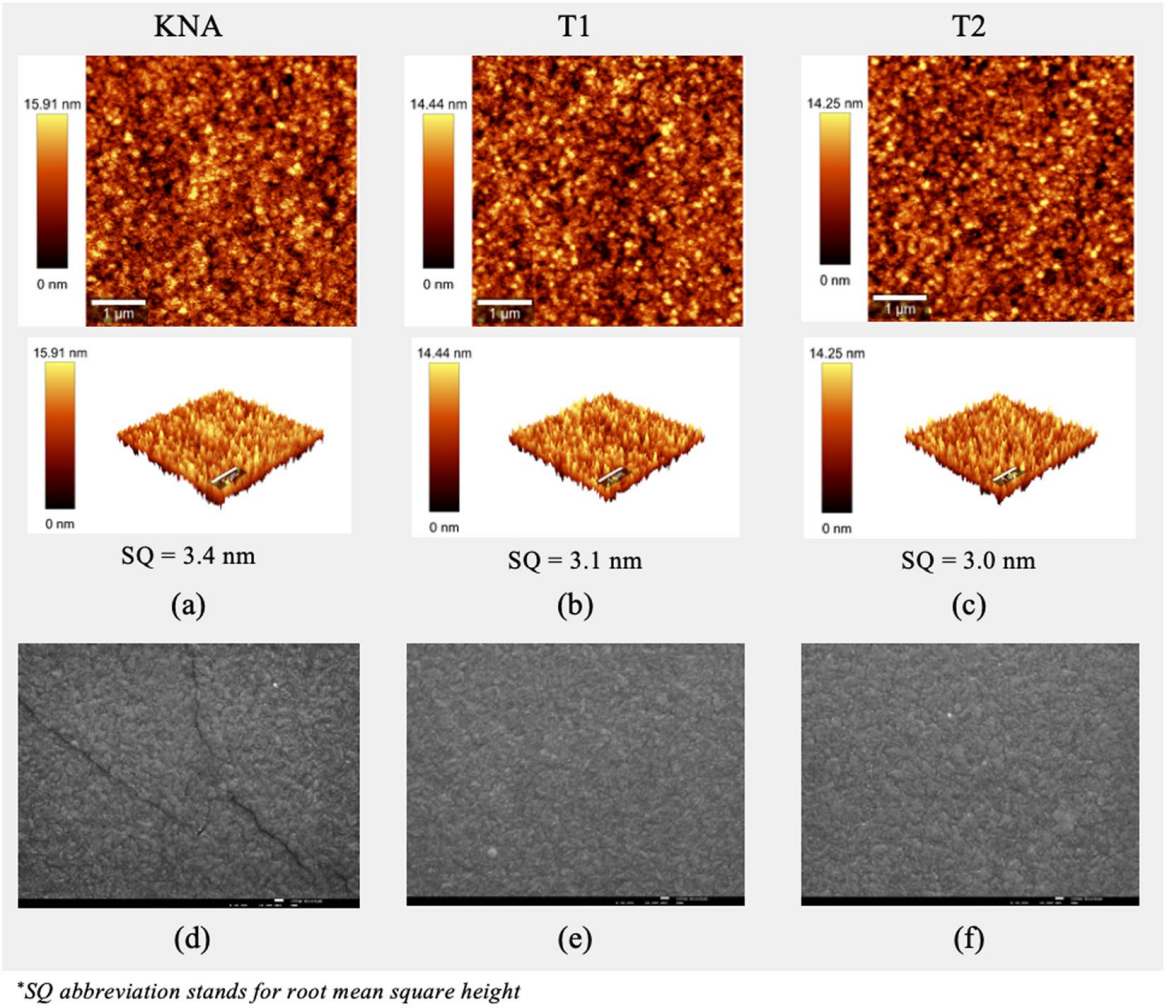
SEM-SEI and AFM images of 460 nm thick AZO films deposited onto Kapton CS substrates with and without (KNA) plasma treatment with He/O_2_ (T1) or H/N_2_ (T2).

The morphology of AZO films deposited onto 25 μm thick Kapton CS were assessed by SEM and AFM. The AFM images, shown in Fig. 2(a–c), clearly indicate that the films roughness is essentially unaffected by the plasma treatment, with rms roughness values of 3 nm. However, the cracks observed in films grown on Kapton substrates are not observed in substrates with plasma treatment with either He/O_2_ (T1) or He/N_2_ (T2) as shown in the SEM images of Fig. 2 (cf. Fig. 2(d) vs. Fig. 2(e,f)). Therefore, one may conclude that the plasma treatment is beneficial for films adhesion and consequently for electronic properties uniformity and enhancement.

### Influence of UV-light

**Figure 3:**
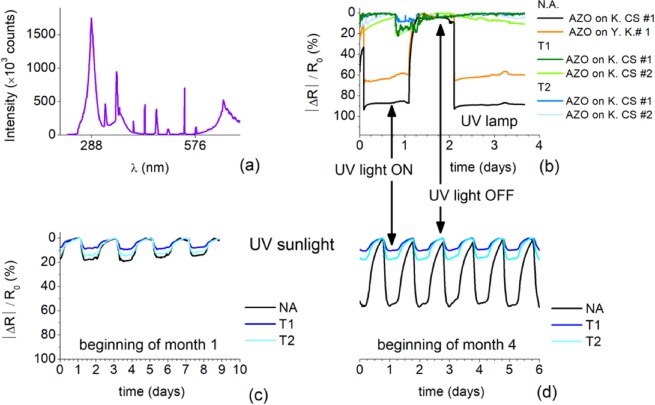
(**a**) Emission spectrum of the UV lamp used. (**b**) Resistance variation of AZO films deposited on Kapton CS (K.CS) and yellow Kapton (Y. K) without (NA) and with (T1-H_2_/O_2_ and T2-He/N_2_) substrate plasma treatment as UV-lamp is tuned ON and OFF. (**c**,**d**) Resistance variation of films grown on Kapton CS measured in two different time windows during sun-light exposure.

Figure 3 shows the variation of the AZO films electrical resistance over time, for different time spans and films; these are expressed as $|R(t)-{R}_{0}|/{R}_{0}$, where *R*_0_ and *R* are the film resistances measured respectively before exposure to the UV-light and at time $t$. The R_0_ values for thin and thick films vary in the ranges 300–650 Ω and 50–200 Ω, respectively. The UV spectrum of the lamp used is shown in Fig. 3(a) displaying a dominant emission peak at the wavelength of 288 nm. Upon UV exposure Fig. 3(b) shows that the films resistance drops, remaining low while UV is ON and recovering to the original value *R*_0_ as UV is switched OFF. This photo-response to UV-light is well reported for AZO films^18^ and it is attributed to the combination of two phenomena: photoconductivity and oxygen photo-detachment. When an electron-hole pair in conduction and valence bands is generated following the UV-photon absorption, supply of photo-generated electrons to adsorbed oxygen can also promote photo-detachment thereby liberating trapped electrons, the electrons trapped at these oxygen sites, that add to the photocurrent. Therefore, both effects may cause an increase in the photocurrent and thereby, a decrease in the baseline resistance, as well reported in the literature for ZnO films doped with Al and with a band gap of 3.4 eV. ALD deposited AZO films revealed to have the same band gap, and as such those effects may also explain the photo response of ALD AZO films. Figure 3(c) shows the effect of UV sunlight on the electrical resistance of AZO films deposited on Kapton CS with plasma treatments (T1 and T2). The resistance variation is more pronounced for AZO films on substrates without plasma treatment The influence of plasma treatment on the films resistance along time is shown in Fig. 3 from (c) to (d). Initially, the response to UV light of AZO films on substrates without and with plasma treatment is similar, being the resistance variation about ∼10% and 20% of the reference, but after 4 months, the resistance variation levels of films on substrates without plasma treatment changed up to 60–80%. Therefore, the results indicate that treatment of Kapton substrates by He/O_2_ or H/N_2_ plasmas enhance the long term stability of the UV-triggered variation of electrical resistance. This maybe related with the fact that, plasma treatment promotes a better interaction of ALD precursors with the substrate surface leading to better adhesion and seed layers from where ALD films growth with improved lattice crystallinity. Therefore, the virgin, untreated, Kapton substrates may have a smoother surface compared to the plasma treated surfaces providing lower adhesion to the AZO films. Across this weaker binding interface, there should develop a larger discontinuity in electronic density that may introduce localized defect states (“surface-like” electronic states) that potentially absorb UV photons and affect the absorption spectra. The resulting enhanced UV absorption is a source of photo-electrons injection in the conduction band, increasing the photocurrent and causing larger baseline variation.

In ZnO the increase in photo-induced carriers due to the UV-light exposure contribute to the thermoelectric transport^19^, as such the electrical resistance, Seebeck coefficient, power factor and power curves for thin and thick AZO films was measured before and during UV illumination.

**Figure 4:**
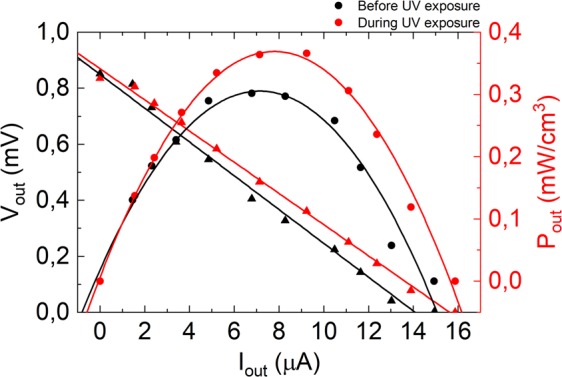
I-P and I-V curves measured for thick films deposited on Kapton CS plasma treated with He/O_2_ (T1), before and during UV-light exposure.

The Seebeck coefficient (S) is not affected by plasma treatment or UV exposure, but is enhanced from ∼75–80 μV/K to ∼105–150 μV/K, when the film thickness is changed from ∼100 nm to ∼460 nm, respectively. Likewise, the power factor (proportional to the square of the Seebeck coefficient) also increased, from ∼71 μW/mK^2^ to ∼302 μW/mK^2^ (as shown in Fig. S1). Such behaviour is the opposite to that observed for AZO films deposited by sputtering^20^, which may be due to the enhancement of conductivity and phonon scattering in the ALD films. With increasing films thickness more phonon disperse at the Al_2_O_3_ interlayers and thereby, the Seebeck coefficients increase. The lower resistance of thicker films leads to higher currents and power outputs as shown in Fig. 4. This illustrates the current-power (I-P) and current-voltage (I-V) curves for thick (Fig. 4) films grown on Kapton CS substrate with He/O_2_ plasma treatment (T1). During UV exposure the films resistance decreases and as a result the power density output increases, specially for thicker films as shown in Table 1. This means that a photo effect is added to the thermoelectric effect, as reported for ZnO crystalline material^19^. Table 1 shows the resistance, the Seebeck coefficient and the power factor variation with ON/OFF UV light exposure for thin and thick AZO films deposited on Kapton CS substrates plasma treated with T1.

**Table 1:** Resistance variation, Seebeck coefficient and power factor of thin (100 nm) and thick (460 nm) AZO films under different UV-lighting conditions.

		100 nm thick AZO on K-CS w/ T1	460 nm thick AZO on K-CS w/ T1
$|\Delta R/{R}_{0}|({\rm{ \% }})$		25	35
$| S| \,(\mu V/K)$	B	79	123
	D	77	107
$PF\,(\mu W/m{K}^{2})$	B	71	276
	D	90	319

B: before | D: during

### Influence of humidity

**Figure 5:**
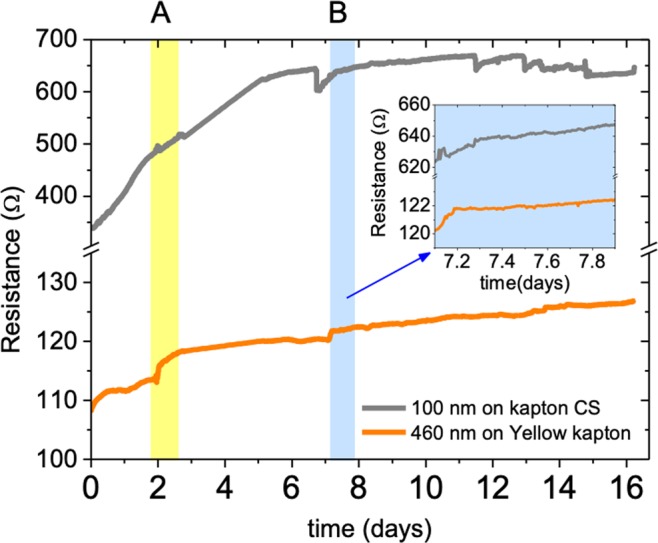
Time dependence of the resistance of 100 nm thick AZO film deposited on Kapton CS (grey curve) and of 460 nm thick AZO film deposited on Yellow Kapton (orange curve). The atmosphere’s humidity was changed for the time periods marked (**A**,**B**). The inset shows the resistance variation for time interval (**B**).

**Figure 6:**
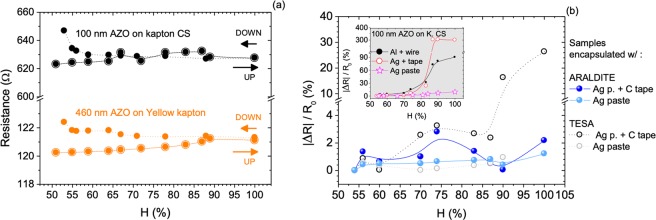
Relative humidity dependence of the resistance of AZO films measured before (**a**) and after (**b**) samples’ encapsulation. In (**a**) Al-evaporated electrodes, (**b**) Al-evaporated electrodes with Al wire attached, Ag paste electrodes with (C. tape) and without conductive tape were tested.

The influence of humidity on AZO films deposited on flexible substrates was evaluated by monitoring the films resistance over time in the dark while using different electrode materials (*e*.*g*. Al and Ag) and changing relative humidity. Figure 5 shows over 15 days the resistance variation of 100 nm and 460 nm thick AZO films grown on transparent Kapton CS and yellow Kapton. During that period of time, the humidity was made to vary from 51% to 100% in two short periods, A and B, and left otherwise constant. In general, the films resistance tends to increase in a first period after which it stabilizes regardless of the humidity changes applied over periods A and B. The result is illustrated in the inset of Fig. 5 for period B (it was similar for period A). The variation of the AZO films resistance as a function of the relative humidity (RH, %) for the particular period B is illustrated in Fig. 6(a) for the upward and downward cycles, using thermally evaporated Al contacts. The global change in resistance is small in all cases, about 2% and 4% for the AZO films (460 nm thick) deposited on yellow Kapton and on Kapton CS (100 nm thick), respectively. The Seebeck coefficient measured before and after the humidity tests also varied only slightly, about 2%, which means that the power factor remained approximately constant for thin and thick films. However, as the inset of Fig. 6(b) shows, other electrode materials (brushed Ag paste, brushed Ag paste topped with conductive tape, or evaporated Al pads with attached Al wire) behave differently with the increase of relative humidity; in particular above RH 75%, resistance increases rapidly, by more than 3-fold for a RH > 90%. This may be related with possible water condensation since the original values of resistance of the films can be almost fully recovered after drying the samples. Compared to the evaporated Al contacts, Ag paste electrodes also provide relatively stable resistance values when moisture is varied. Furthermore, the sensitivity to humidity of any of the tested electrodes can be reduced by protecting the thin films and electrodes. The protecting materials considered for transparent, flexible and large area device applications need: (1) not hinder bendability, (2) be of low cost and easy implementation, (3) be applicable in large areas and compatible with mass production and (4) not significantly affect the transparency of the films. Transparent TESA adhesive tape (ref. 61562^21^) 25 μm thick and ARALDITE epoxy adhesive were selected to test as they match the mentioned conditions. As shown in Fig. 6(b) encapsulation with these materials allowed keeping the films resistance changes within 2% in most of the cases, even when electrodes highly sensitive to humidity are used (to ascertain of the adequate transparency of the protective coatings refer to Fig. S2 which allows comparison of the transmittance spectra of AZO films on yellow Kapton with and without protecting layers).

### Bending tests

**Figure 7:**
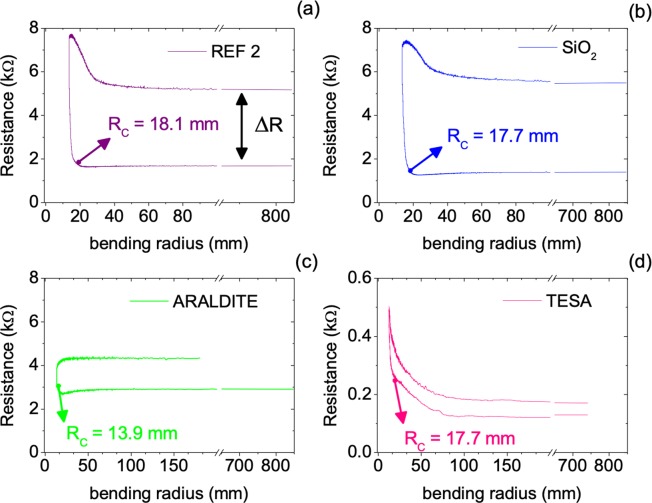
Continuous resistance profiles along 1 cycle for a reference 460 nm thick AZO film without encapsulation (**a**), and with encapsulation of SiO_2_ (**b**) Araldite (**c**), and TESA (**d**).

**Figure 8:**
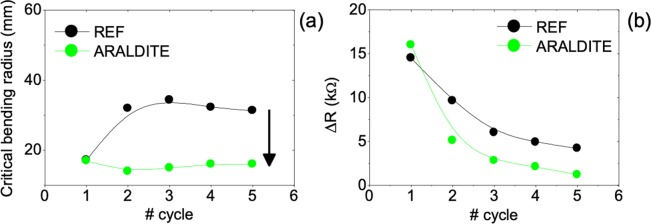
Plots of the behaviour of the critical bending radius *R*_c_ (**a**) and resistance variation (**b**) as from 460 nm thick AZO films as a function of the number of bending cycles.

As shown above, ALD thick films provide superior thermoelectric performance and are thereby preferred for integrating many large area thermoelectric applications. An example is a smart window that make use of AZO thermoelectric modules deposited on a curved substrate^22^. Hence, characterizing the flexibility and stability of AZO films in this type of applications is important. Herein, a home-built bending test machine was used to characterize 460 nm thick AZO films deposited on the low cost Yellow Kapton substrate with and without encapsulation, through *in-situ* resistance measurements (Fig. S3). All measurements were performed according to the International Electrotechnical Commission standard 47/2199/NP draft^23^ and are depicted in Figs. 7 and 8.

Figure 7 displays the electrical resistance measured as a function of changing radius of curvature over one full cycle for samples without encapsulation and with encapsulation of SiO_2_, ARALDITE and TESA protective coatings (one full bending cycle corresponds to the sample bending in the forward direction followed by relaxing in the backward direction, regaining the starting flat position). The critical bending radius *R*_c_, is the radius of curvature below which the resistance exceeds 5% of its original value (*R*_0_) as illustrated in Fig. 7. The first observation is that *R*_c_ of encapsulated AZO films is smaller than the *R*_c_ of uncoated samples, indicating that the protective coatings selected do positively affect the bendability of the films. From those, ARALDITE was chosen to make repetitive bending tests, as it produced the lowest critical bending radius *R*_c_. Moreover, SEM images (in Fig. S4) of Fig. 7 samples surface show that the reference sample has visible damage at the bending area, i.e. several linear wrinkles, as encountered in the sample coated crystalline SiO_2_ but to a much smaller extent. The surface of the sample coated with TESA exhibited bulges that resemble bubbles that may have been created in certain areas due to peeled off caused by bending. In contrast, the SEM image of the film coated with ARALDITE shown a very smooth and homogeneous coating.

Figure 8 shows the behaviour of the critical bending radius (a) and electrical resistance (b) as function of the number of cycles performed in samples with and without ARALDITE protective coating: it clearly indicates that the resistance varies significantly in the first few cycles, the first two or three, remaining rather constant thereon (samples resistance profiles measured at each bending cycle are shown in Fig. S6). The change is smaller for the sample covered with ARALDITE. As for the critical bending radii the behaviour can be understood considering that most of the damage develops immediately during the first cycle, most probably in the form of extended defects that collapse as micro-cracks capable of interrupting electrical paths on subsequent bending (provided that no inhibiting action exists): in uncoated samples further bending-unbending cycles open and close these micro-cracks, effectively breaking and (re)making a number of conductive paths (akin electrical switches), thus influencing the measured resistance and appearing as an increased critical radius. On the contrary, in coated samples the protective coating can act to force these micro-cracks to remain closed for longer, hence more conductive paths (or electrical switches) to keep closed for longer upon bending, allowing for smaller critical radii. Further cycles would not change the picture dramatically, although higher levels of point like defects would result, justifying a progressive increase of the overall resistance (due to enhanced scattering and/or trapping of charge carriers) and, as such, a decrease of the resistance change as observed. In the limit a near zero change in resistance would be expected. With this in mind, a new set of samples were tested and repeatedly bent over 20 cycles, while varying the curvature radii out of the critical range. The samples were bent forward and backward up to 100 mm bending radii. The change in resistance (*ΔR* = *R*_*f*_ − *R*_0_) was around zero for all samples. The surface morphology of encapsulated samples after 5 and 20 cycles is shown in the SEM images of Fig. S5.

## Conclusions

This work demonstrates that AZO films deposited on flexible substrates retains good thermoelectric properties with great stability under humidity environment and bending. Therefore, ALD is a viable and practical technology to produce AZO films consistently and reproducibly with the mechanical and electrical properties that make these suitable for large flexible transparent thermoelectric generator applications.

The work further demonstrates that plasma treatment of Kapton substrates, with He/O_2_ (T1) or H/N_2_ (T2), prior to the atomic layer deposition (ALD) of Al doped Zn oxide (AZO) films enhances the stability over time of their electrical and thermoelectric properties, and reduces films cracking in subsequent handling. The highest Seebeck coefficient (∼120 μV/K) and power factor (∼300 μW/mK^2^) were obtained for 460 nm thick AZO films, and the power output density for a small volume element of these films (3 × 6 mm^2^ of area and ∼460 nm of thickness) and a temperature difference of 10 °C is ∼0.3 mW/cm^3^. This increases to 0.4 mW/cm^3^ during exposure of the films to UV-light, due to the photo-thermoelectric effect. The electrical resistance of the films proved to be stable under humidity, even when exceeds 75%. Nevertheless, it was shown that electrodes moisture absorption and retention can be avoided through their covering with a protective layer such as TESA and ARALDITE. These are highly transparent keeping the original transmittance of the AZO film in the visible wavelength range and did not increase the films critical bending radius, which in the case of ARALDITE it was even further reduced, from 19 mm to 14 mm. Continuously bending of films down to 100 mm over 20 cycles, led to films resistance variations near zero, demonstrating the films durability under mechanical stress. These observed changes in critical bending radii and electrical resistance upon repeated cycling may be explained by a proposed mechanism, based on the immediate generation of extended defects that collapse to micro-cracks capable of cyclically interrupting charge transport, along with continuing increase in point like defects which upon scattering and/or trapping charge carriers increase the overall resistance.

## Supplementary information

Supplementary Information
