# Supplementary material for: Stability under humidity, UV-light and bending of AZO films deposited by ALD on Kapton
Source: Sci Rep. 2019 Nov 29;9:17919. doi: 10.1038/s41598-019-54451-0 (PMC6884584; doi:10.1038/s41598-019-54451-0)
Supplement: Supplementary file 1 — Supplementary Information [file 41598_2019_54451_MOESM1_ESM.docx]

**SUPPLEMENTARY INFORMATION**

**Stability under humidity, UV-light and bending of AZO films deposited by ALD on Kapton**

A.C. Marques^1*^, J. Faria^1^, P. Perdigão^1^, B. M. M. Faustino^1^, Riina Ritasalo^2^,
Katiuscia Costabello^3^, R.C. da Silva^4^, I. Ferreira^1^

1) CENIMAT/I3N, Departamento de Ciência dos Materiais, Faculdade de Ciências e Tecnologia, Universidade Nova de Lisboa, Caparica, 2829-516, Portugal

2) Picosun Oy Masalantie 365, FI-02430 Masala, Finland

3) GRINP SRL, via De Francisco 123, 10036 Settimo T.se (TO)

4) IPFN-IST/UL, Instituto de Plasmas e Fusão Nuclear, Instituto Superior Técnico, Universidade de Lisboa, Estrada Nacional 10, 2695-066 Bobadela, Portugal

* e-mail: acl.marques@fct.unl.pt

Figure S1 shows the plot of the thermo-voltage (ΔV) versus applied temperature difference (ΔT) as measured from 100 nm and 460 nm thick AZO films deposited on Kapton CS, with planar Al electrodes with a size of 3×6 mm^2^ and separated by 3 mm. The Sebbeck coefficient (S) extracted from the slope of the linear fit, together with the electrodes dimension, film thickness and its electrical resistance (R) enabled the calculus of the power factor through the equation illustrated in Fig. S1-a).


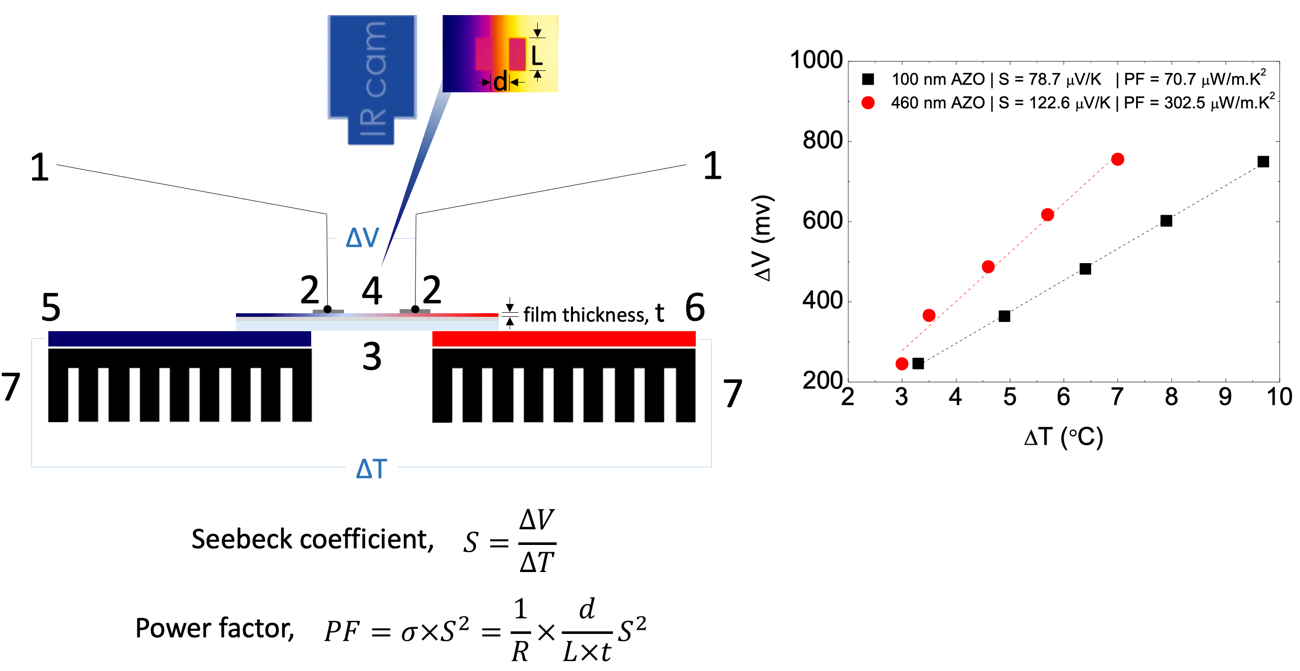


**Figure S1** – (a) A schematic of the cross-sectional view of the home-made apparatus for the Seebeck measurements of thin films. (1) Nano-voltmeter probes to measure the thermo-voltage; (2) electrodes on thin film; (3) Kapton substrate; (4) Thermoelectric thin film; (5) Cold Peltier; (6); Hot Peltier; and (7) heat-sink. (b) Thermo-voltage vs. temperature plot as measured from thin (~100 nm) and thick (~460 nm) AZO films deposited on Kapton CS. From the linear fit slope the Seebeck coefficient was determined and enabled the calculus of the power factor, being both values indicated in (b).

The transmittance spectra of 460 nm thick AZO film on yellow Kapton without (REF) and with a protective covering of TESA, ARALDITE and SiO_2_ are shown in Fig. S2. All are highly transparent and do not deteriorate the original transmittance of the AZO film in the visible wavelength range.


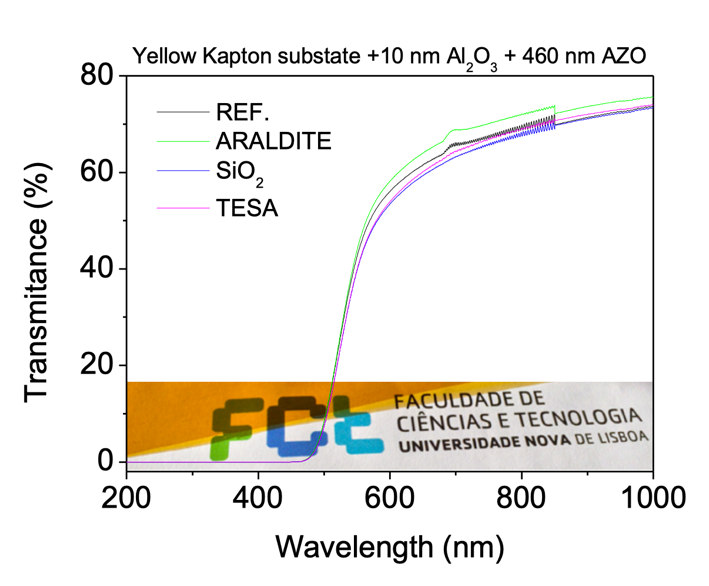


**Figure S2** – (a) Optical transmittance spectra of thick AZO films deposited on Yellow Kapton before (“REF.” sample) and after applying Araldite adhesive, TESA adhesive barrier tape and SiO_2_ (deposited by RF sputtering) protective layers.

The set-up developed in house is Arduino controlled and it is shown in the photos of Fig. S3. The Arduino boards are used to move the DC motor and to readout the electrical resistance at each bending radius. The central photo shows the sample on its starting flat position and the photo in the top left corner shows the sample bending for the critical bending radius. The calculus of the bending radius is performed by means of the equation in Fig. S4 for each length variation ($dL$). In the equation, L and $dL/L$ denotes the initial length of the sample and the rate of chance of the length, and $h$ is the sum of thicknesses of the substrate and of the film.


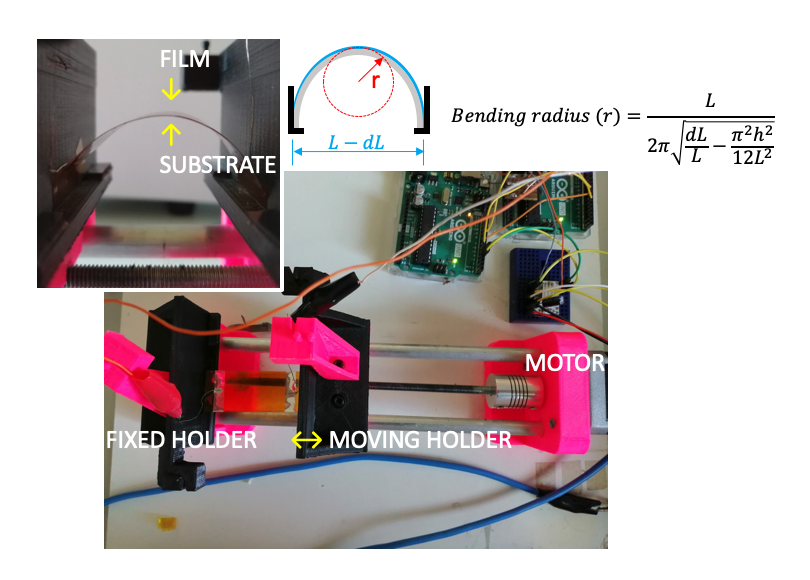


**Figure S3** – Home-built bending tester set-up in compliance with International Electrotechnical Commission standard 47/2199/NP draft. The sample is shown on the flat position and for the critical bending radius (top left corner photo).

Figure S4 show the surface of uncoated and coated AZO films after one bending cycle, performed to determine the critical bending radius. The reference sample (named REF2) shows significant damage (linear wrinkles) as the sample coated with crystalline SiO2 but to a smaller extent. TESA coating show bulges resembling bubbles that may have been created due to coating peeling off in certain bent areas. In contrast Araldite shows a very smooth and homogenous coating without visible defects. This was selected to make repetitive bending tests.


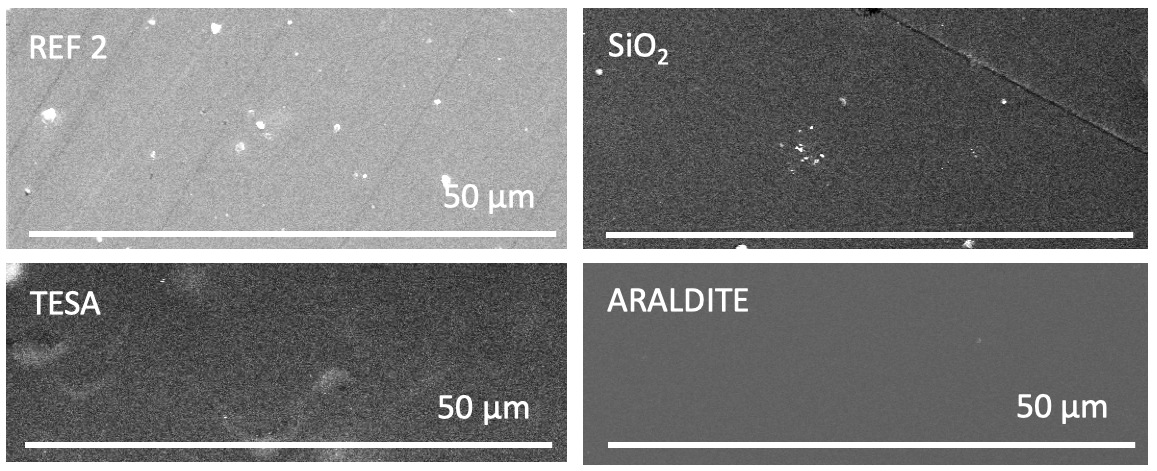


**Figure S4** – SEM images of an un-coated AZO film and coated AZO films with sputtered SiO_2_, TESA and ARALDITE.

Figure S6 shows the resistance profiles of two samples of 460 nm thick AZO films on yellow Kapton without and with ARALDITE covering, measured along 5 consecutive bending cycles.

**Figure S5** – Continuous resistance variation while bending an AZO sample without – REF (a) – and with an ARALDITE protective layer (b) over 5 consecutive cycles.

Figure S6 shows SEM images from the surface of coated AZO films with SiO_2_, TESA and ARALDITE after 5 and 20 repeated bending cycles up to a curvature radius of 100 mm (higher than the critical radius). For the second set of cycles fresh samples were used. The surface areas shown in Figure S6 correspond to a small section of the bended area. After the 5 cycles, the surface of the samples encapsulated with SiO_2_ show smooth linear wrinkles that strengthen after 20 cycles. The wrinkles or ‘bubbles’ observed at the surface of TESA samples after 5 and 20 cycles, respectively, may be due to its dethatch in certain areas, and the type of defects arising following bending may significantly vary from sample to sample. After 20 cycles ARALDITE coating seems to suffer damage. However, ARALDITE wrinkles should not be deep because electrical performance was not compromised.


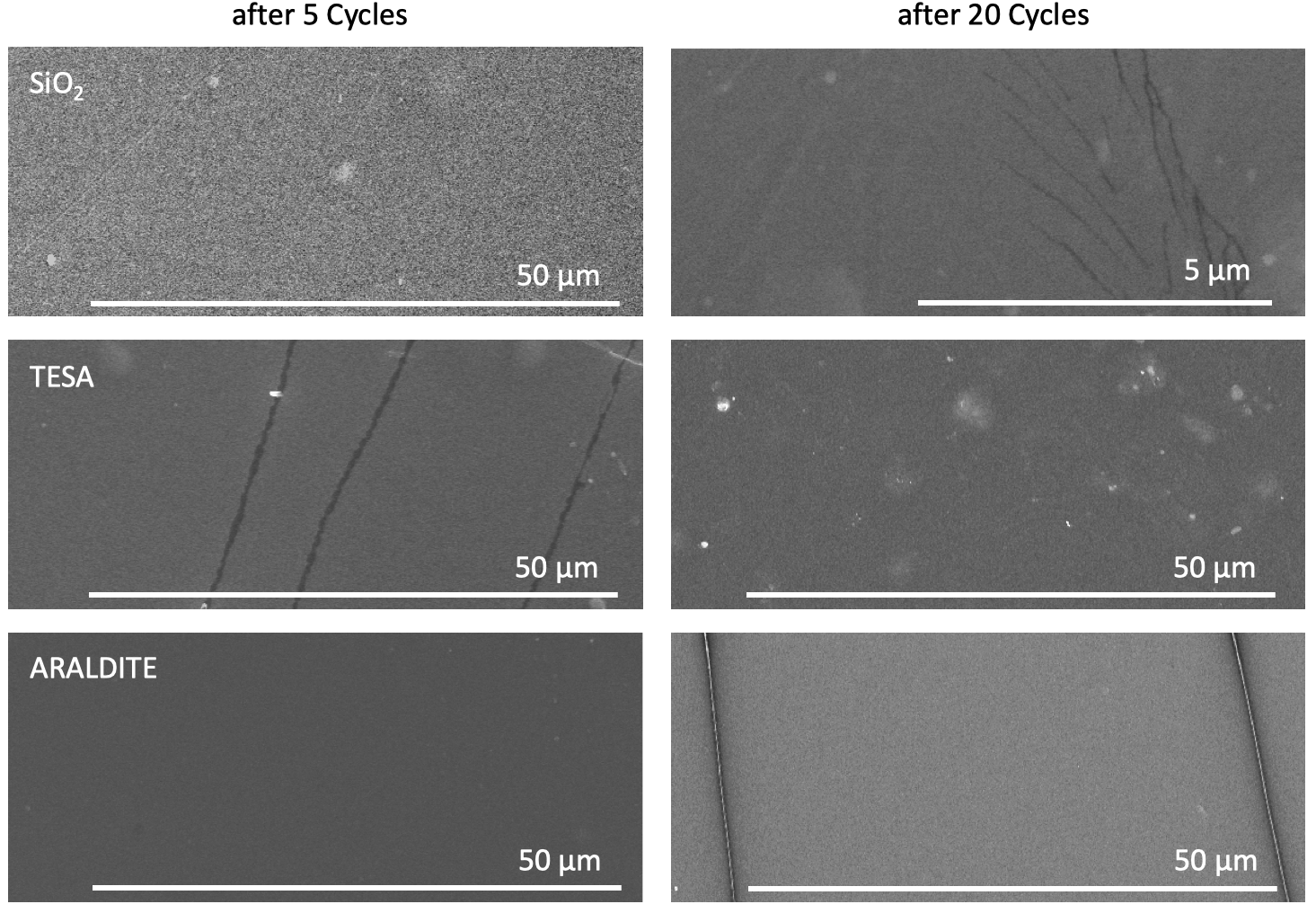


**Figure S6** – SEM images of encapsulated AZO films after 5 and 20 cycles. For the second set of cycles new samples were prepared.
